# The Challenge of Sustainability of High-Cost Oncological Drugs: A Budgeting Model in an Italian Cancer Center

**DOI:** 10.3390/ijerph182413413

**Published:** 2021-12-20

**Authors:** Carla Masini, Davide Gallegati, Nicola Gentili, Ilaria Massa, Raffaella Ciucci, Mattia Altini

**Affiliations:** 1 Oncological Pharmacy Unit, IRCCS Istituto Romagnolo per lo Studio dei Tumori “Dino Amadori” IRST s.r.l., via Piero Maroncelli 40, 47014 Meldola, Italy; carla.masini@irst.emr.it; 2 Budget and Planning Unit, IRCCS Istituto Romagnolo per lo Studio dei Tumori “Dino Amadori” IRST s.r.l., via Piero Maroncelli 40, 47014 Meldola, Italy; davide.gallegati@irst.emr.it; 3 Health Management, Data Unit, IRCCS Istituto Romagnolo per lo Studio dei Tumori “Dino Amadori” IRST s.r.l., via Piero Maroncelli 40, 47014 Meldola, Italy; 4 Outcome Research Group, IRCCS Istituto Romagnolo per lo Studio dei Tumori “Dino Amadori” IRST s.r.l., via Piero Maroncelli 40, 47014 Meldola, Italy; ilaria.massa@irst.emr.it; 5 Independent Researcher, 00100 Rome, Italy; raffa.ciucci@gmail.com; 6 Health Direction Unit, Azienda Unità Sanitaria Locale della Romagna, via De Gasperi 8, 48121 Ravenna, Italy; mattia.altini@auslromagna.it

**Keywords:** oncology, budget, process, sustainability, economy, effectiveness, oncology pharmacy

## Abstract

In Italy, drug expenditure governance is achieved by setting caps based on the percentage increase in hospital spending compared to the previous year. This method is ineffective in identifying issues and opportunities as it does not consider an analysis of the number of treated cases and per capita consumption in local and regional settings. The IRCCS (Scientific hospitalization and treatment institute) Istituto Romagnolo per lo Studio dei Tumori (IRST) “Dino Amadori” in Meldola, has developed and adopted an effective management model designed to oversee pharmaceutical expenditure, guarantee prescription appropriateness and quality of care to patients. The budget setting follows a structured process which evaluates determining factors of the expenditure such as expected patients calculated according to the epidemiology and to national and regional indications of appropriateness, mean cost per patient calculated on the average period of demonstrated efficacy of the drug and use of drugs with the best cost-effectiveness ratio. Strict monitoring and integrated purchasing processes allow for immediate corrective actions on expenditures, as well as a continuous dialogue with the region in order to guarantee consistent funding of IRST activities. The model, presented in this article is efficient and implements concepts beyond the conventional “silos” approach and national and regional governance tools, in terms of patient centricity.

## 1. Introduction

Recently, attention in pharmaceutical governance has focused on hospital channel expenses (c.d. direct purchases, i.e., drugs purchased by the health facilities of the NHS (National Health Sistem), including class-based drugs in direct distribution and on behalf of, considered as distribution, directly to patients for use at home). This type of expense has grown more than the gross cost for class A drugs (drugs paid for and distributed by the National Health System, not for hospital use only) distributed directly to patients by hospital pharmacies or through territorial pharmacies (direct purchasing), more than a quarter of which are oncology drugs (26% in 2019).

Pharmaceutical expenditure represents a significant part of the resources that the Italian government annually spends on healthcare (EUR 23.5 billion in 2019 approximately 21.4% of FSN, National Healthcare Fund [1]).

In Italy, according to the logic of a silo budget, the national cap for pharmaceutical expenditure is defined as a percentage of total funding for health expenses (National healthcare fund—FSN), supported by a rigid monitoring mechanism to ensure compliance and contain overspending [2]. These are mostly represented by paybacks, supported by more sophisticated mechanisms such as MEAs (Managed Entry Agreements) with much lower impact.

The total percentage of pharmaceutical expenditure on the national health fund remained at 14.85%, but the territorial cap was reduced to 7.96%. Fluctuations in hospital expenditure for drug purchase impact more than territorial variations, confirming the need for more stringent governance and monitoring [3].

**Figure 1:**
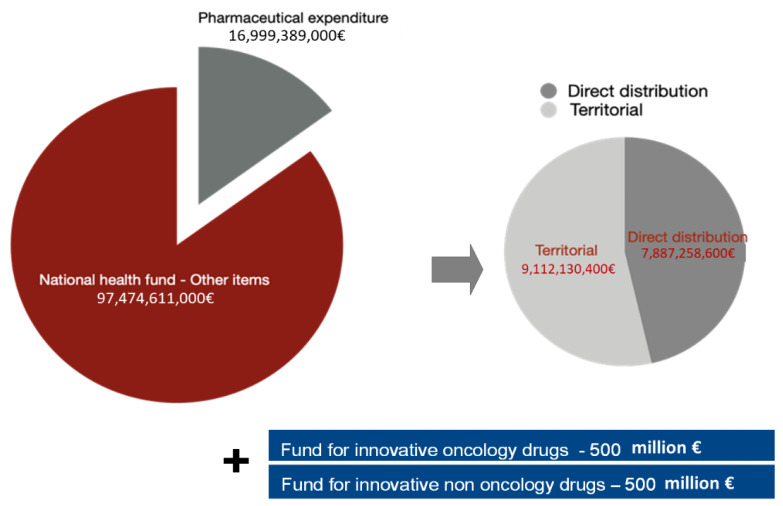
Composition of pharmaceutical expenditure and national health fund (NHF).

Two additional Funds were also established in 2017, each with a maximum expenditure of EUR 500 million dedicated, respectively, to innovative oncological and non-oncological drugs [4,5] (Figure 1). 

New rules and regulations concerning the switching to biological drugs with their biosimilars and the purchase of biological drugs with expired patents were also established [6]. The above-mentioned tools generated a silo approach.

Pharmaceutical expenditure for direct purchases has grown constantly beyond the established cap, despite the tools implemented at a national level, maintaining the already present state of fundamental structural under-financing. The monitoring report by the Italian Medicines Agency (AIFA) for January–December 2019 showed an over-expenditure of 6.89% above the target, which translated into a deviation, in absolute terms, of approximately 2.6 billion euro, equal to 9.02% on the NHF [1]. In 2018, the same monitoring report by AIFA showed an overall overrun of €2.2 billion with a total impact on the NHF of 8.85% [7]. This overshoot was evident, although in different proportions, in all Italian regions. The innovative oncological drugs fund, established in 2017, was initially underused (402 million euro in 2017), but in 2018 recorded a strong increase due to the overall under-financing of hospital pharmaceutical expenditure (over 600 million euro in 2018) and the continuous increase in expenditure for cancer drugs in the EU [8].

This Italia system to contain healthcare and pharmaceutical spending generates a silo model, that is, a model managed through expenditure caps that are not communicating with each other. One of the major limitations of this system is that it does not allow those who administer the health facilities to merge any residual funds into the other items. The IRST of Meldola created a budgeting model presented in the article, and that represents one of the many ways of dealing with a silo system and respecting national sales cap and obligations.

The aim of the article is to propose a method that allows programming resources close to reality, overcoming the limits of planning only using historical data without consistency with foreseeable changes in the scenario such as the availability of new medicines, the increase in financial resources and more, with the use of tools available at the national level. 

The article describes the complex process of planning the financial resources of IRST and its strengths, such as integration with the quality levels of care and involvement and sharing with all parties. Each step is explained, and the results are discussed through tables and figures useful to reproduce the model if desired.

The article starts from describing the methods of allocating the budget at regional level and the tools used to then move on to the IRST model. Methods are represented from the strategic point of view and then tools are described.

## 2. Materials and Methods

### 2.1. The Regional Method

Regional and local settings have often highlighted the limits of these funds, both in terms of capacity as well as resource allocation to the regions. Resource allocation is often defined in line with historical data but does not consider regional epidemiological characteristics or per capita costs, which are widely used in the analysis of pharmaceutical expenditure.

All regions focus on three different management circumstances:

1. authorization for drug prescription;
2. drug purchase;
3. management of the prescription process and governance of appropriateness.

Most Italian regions have adopted the PTR/P (Regional/Provincial Therapeutic Handbook) or CTR (Regional Therapeutic Commission) as binding guidelines for drug purchase.

The Emilia Romagna Region (RER), in addition to the tools used for all drugs, has implemented additional measures with further, restrictive indications for oncological drugs. The Regional Group of Oncological Drugs (GReFO) is a multidisciplinary group that conducts a transparent, reproducible and flexible evaluation process of drugs using a value-based approach according to the Grading of Recommendations Assessment, Development, and Evaluation system (GRADE) [9,10,11]. GRADE uses evidence and robustness of efficacy data with the aim of supporting the definition of the degree (strong/weak) for each recommendation provided. The group also publishes useful documents for clinicians on appropriate drug use and for local healthcare local administrations (ASL) on the governance of pharmaceutical expenditure. These provide a basis for sharing therapeutic choices among health professionals with the aim to support correct drug placement in therapy and indications on the expected use (overall and specific per pathology) in an epidemiologically homogeneous population. The indications of the GReFO are also a valid monitoring tool to support the governance of pharmaceutical expenditure in terms of comparability and identification of “unwarranted variations”.

In order to ensure correct funding of oncological drugs to local patients, the RER has set up an additional fund (Fund B) [12], which includes innovative AIFA oncological drugs with expired innovation designation, additional therapeutic indications of innovative drugs on the AIFA list (potential innovation or non-innovation) and innovative potential or non-innovative high-cost oncological drugs. In 2019, Fund A financed an approximately 5.1-million-euro expenditure, and the AIFA supported Fund B by approximately 5.6 million euro. 

During the negotiation, RER assigns the total budget of Funds A and B to innovation in oncology, defines regional spending objectives and negotiates with each single hospital target for direct purchase, calculated as percent variation in the budget of the previous year. Choice of funding for innovative non- and oncological drugs (Fund A) and Fund B, though, is based on the GReFO indications.

In order to more correctly reflect the real case scenario in the region and facilitate negotiation, RER applies forecast criteria similar (with intrinsic differences) to those used at IRST Meldola. Indeed RER and IRST Meldola use the same databases (DBO—Oncological Database [13]); AFO—Hospital Pharmaceutical Assistance—and FED—Total reimbursed expenditure [14]) for budget forecasts, yet the differences which govern an oncological research hospital have to also consider other factors.

The number and duration of pharmacological clinical trials conducted at IRST and different evaluation in terms of costs of treatment (correct allocation of costs for treatments with a progression-free survival (PFS)), will have a major impact both on expected and planned expenditure, which can lead to an underestimation/overestimation of costs and consequent overrun.

In such a complex environment of differential budget constraints, there is the need to develop a tool that allows resources to be managed more consistently with health needs. In this paper, we present our budgeting and management model as an answer to track spending on cancer drugs. 

### 2.2. The Hospital Context of Services

The IRST (IRCCS Istituto Romagnolo per lo Studio dei Tumori “Dino Amadori”—IRST S.r.l.) based in Meldola is an oncological, scientific hospital and treatment institution with a territorial engagement (400,000 residents in its territory of competence) in which pharmaceutical expenditure represents approximately 30% of the total budget. In 2019, expenditure was approximately 27.8 million euro (excluding medicinal gases and blood derivatives): this represents 3.4% of regional expenditure for direct purchases.

The IRST is a public–private partnership, with special focus on cancer in its dedicated oncology ward, three outpatient day hospital/day services located at three different operational sites in Meldola, Forlì and Cesena. It has two departments of Radiotherapy, in Meldola and Ravenna, a Radiology department, a laboratory of bioscience and an oncological pharmacy. The Meldola site provides a complete clinical coverage for patients offering day hospital and day services/outpatients services for the administration of intravenous drugs, radiotherapy and radiometabolic medicine. In 2019, the IRST managed about 28,000 patients, including 5000 with pharmaceutical-based therapies.

The governance strategy regarding pharmaceutical expenditure developed in this setting consists of a structured budgeting control process and an innovative forecasting model. The latter combines estimates of the number of patients who can undergo oncological treatments (based on epidemiological data), potential new molecules available, retrospective and prospective evaluation of clinical studies, and the use of drugs in the approved indications as well as their off-label use. An ad hoc “negotiation” phase ensures sharing of common objectives and planned resources with all the parties involved in the activities. To support this process, robust innovative tools are used to monitor the trend of expenditure compared to forecast, allow prompt intervention on resources management and ensure compliance with the indicators defined to monitor prescriptive appropriateness of drugs. 

### 2.3. The IRST of Meldola: An Effective Programming Model

#### 2.3.1. Strategic Planning

**Figure 2:**
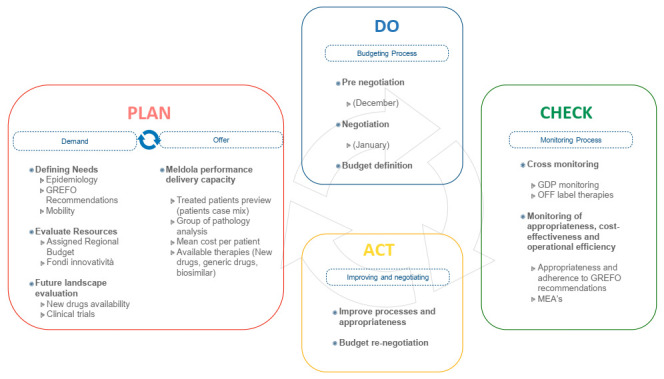
The IRST model.

IRST has a matrix organization in which GDPs (Pathology Groups—simple departmental structures), multidisciplinary team units responsible for following patients throughout their diagnostic and therapeutic pathway and provide for healthcare services in available structures (ward, day hospital, outpatient clinics, etc.), play the central role (Figure 2). 

GdPs prioritize their resources in a patient-centric manner and directly negotiate with the general management on the resources allocated within their oversight in the specific reference pathologies. 

Budgeting in IRST is based on a multidimensional planning scheme divided into performance-based indicators according to a balanced scorecard perspective-based model (customer/patient; internal management; learning and innovation) (Appendix A depicts an example of performance target for each area is presented). Objectives and goals for GdPs and other IRST units are structured on this basis.

The Oncological Pharmacy Department is the key contact point for the management of drugs and medical devices for the entire institution. It is the reference body for negotiation of budget with GdPs, checking the appropriateness of purchases, and monitoring consistency between final expenditure and forecasted budget. The pharmacy department is strongly patient-oriented through GdPs-dedicated pharmacists.

#### 2.3.2. The Complexity of Defining Needs

Identifying specific needs represents the first step for a correct planning of resources. The IRST model is based on a multifactor analysis which considers the following factors on a specific pathology (GDP) basis:

- Anticipated number of patient case-mix to calculate new patients for each line of treatment derived from: Epidemiology of the reference population, GReFO recommendations, Oncological Database (DBO);
- Potential new therapeutic opportunities (new molecules) and efficiency-based opportunities (generic, biosimilar, better cost-opportunity drugs, etc.);
- Ongoing and future clinical trials with high-cost drugs;
- Drug consumption by pathology and number of patients treated (derived from Electronic Health Records (EHR);
- Reimbursement policies regarding early access drugs (according to L.648, Off label use, AIFA fund of 5% [15], CNN [16], UT).

At year-end, the variables are integrated using the previously cited appropriateness criteria [17].

In addition, off-label uses, uses for therapeutic indication of each drug, number of patients treated, the relative number of treatment cycles and average PFS for each treatment are also considered in order to have a complete picture for each GdP. Meldola is also a research hospital and must also consider individualized incident patients and ongoing (prevalent) patient treatment schemes on which to calculate the duration of the therapeutic cycles based on OS (Overall Survival) or PFS (Progression-Free Survival). The institution’s scientific nature makes it a site for numerous sponsored clinical trials which provide a free supply of oncological drugs for treating the enrolled patients. In addition, these trials may allocate funding linked to the activities carried out (grant per patient) and indirectly determine an avoided cost for drugs not used in clinical practice. In 2019, the cost avoided increased compared to the previous year due to an increase in almost all GdPs expenses covered by clinical trial.

To complete an accurate needs analysis, IRST also develops reimbursement scenarios for new drugs or new indications, based on information available within initiatives such as AIFA’s Horizon Scanning. IRST consequently updates the consumption estimates for the various therapeutic areas and completes the definition of needs by November.

### 2.4. Budget Definition

**Figure 3:**
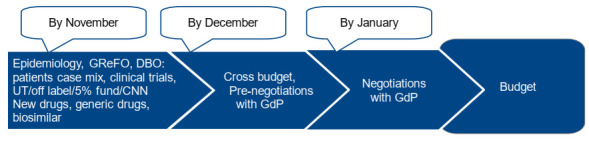
Budget process.

The needs assessment phase is followed by a pre-negotiation phase, during which the Budget Committee (Pharmacy Director, Staff Area Director, DIT Director, UOBSC Director and URTTF Manager, Structural Resources Area Director, Information Technology and Technical Service, Head of URP and Communication, Quality Manager) and the Senior Pathologies leaders (medical doctors in charge of the different simple departmental structures) convene. Achievement of the current year’s objectives is compared with what is defined by the initial negotiations with each operating unit. In this phase, new objectives and the resources needed, including personnel, drugs and devices, are discussed for each GdP. The pre-negotiation phase ends within the month of December (Figure 3).

The subsequent step is the negotiation phase. It involves the GdPs, the budget committee, the strategic direction (General Manager, Scientific Director and Healthcare Director) and the Department heads. In this phase, the proposal drawn up in pre-negotiation is discussed; the budget is consolidated and financial compatibility and consistency with the IRST guidelines and mission are evaluated. This is an important phase of sharing/partaking and integration between management with communication in the assessment of the quality of care provided. In addition, the research budget, typical of an IRCCS, is defined in its aspects: scientific production (impact factor and other indexes), performance for participation in competitive tenders, ability to enroll in sponsored clinical trials, etc. 

The objectives of the GdPs are considered in accordance to appropriateness and drugs cost-effectiveness defined through a set of indicators, rather than with respect to expenditure caps; overshoots/overspending is managed as a signal for an in-depth analysis of the related reasons. Drug-related budget indicators are:

- Prescription appropriateness, considered as percentage, %:
  1. Compliance with the standards of use for pathology settings defined by the GReFO;
  2. Use of cheaper drugs according to GReFO recommendation (Cost-opportunity);
  3. Use of generic and biosimilar drugs;
  4. Use of off-label therapies linked to precision medicine.
- Operational efficiency
  1. Percent deviations;
  2. Stock turnover indices;
  3. Implementation of the drug day/drug month for the preparation of high-cost drugs;
- Quality and safety
  1. Rate of digitalization of the prescription-preparation-administration chain;
  2. Percentage use of robotic preparations.

At the end of this process, within the month of January of the forecast year, the budget is consolidated. 

#### Objectives for Innovative Drugs

During budget allocation, the RER shares with the GdPs the regional estimates and assigns the budget for the annual statements. The methods applied to estimate innovative drug costs mark an important evolution compared to the traditional budgeting methods based only on historical expense evaluation. Despite this, the risk of underestimating or overestimating the final cost still exists as spending is influenced by factors that are difficult to predict, such as variation in the number of patients, uncertain attribution of innovativeness, etc. An underestimation of the real costs can expose IRST to a budget overrun and financial pressure, not directly attributable to inappropriateness, in cases of lack of coverage by the region. To avoid financial pressure, IRST proactively concurred with RER an active feedback mechanism to discuss the budget allocated during the year, with regular evidence-based updates of the activities carried out. 

### 2.5. Monitoring

During the year, continuous monitoring of prescription appropriateness is verified by the pharmacist through an IT tool called “first day first cycle”, which allows an evaluation of therapies within a therapeutic pathway and line of treatment. Each GdP checks all the new therapies required by a physician and confirms them a week/ten days before the treatment administration. If the appropriateness criteria are not confirmed, a dialogue is sought. Furthermore, besides regularly scheduled monitoring activities during the year, additional monitoring is carried out in order to verify all expenditure targets and compliance according to GReFO recommendations in terms of appropriateness and therapeutic adherence. In case of a potential overrun on the forecast by the hospital’s pharmacy, further in-depth indicators are analyzed. If the evaluation confirms the correct delivery of a given treatment, a budget re-negotiation process starts with management control. If, on the other hand, a scenario of repetitive inappropriateness in terms of prescription and budget overrun is identified, the internal group discusses with the Healthcare Administration about corrective actions to be implemented. For example, Healthcare Administration discusses with all parties involved about a therapeutic switch from originator to its biosimilar with a lower cost in specifically defined patients and identifies patients that may proceed to this option.

### 2.6. IT Tools

The availability of integrated IT tools able to guarantee a high level of interoperability is crucial in the monitoring and control context above described. In IRST, the digital medical record, the purchasing and warehouse management software, the production control software for oncological preparations and the administrative-accounting software are all integrated. This includes sharing basic information, such as item master data, between suppliers and departments, in respect of organizational processes. The integrated application platform contains the Enterprise Resource Planning (ERP) software, which manages the entire formal purchase process, the cancer drug preparation software and the medical record that follow the care pathway in all its aspects from diagnosis to treatment administration and drug consumption to prescription, considering also request for drug and health goods, drug formulation, administration of therapies and consumption information. 

The knowledge related to these processes and needed for the budget path is guaranteed by analytical accounting set up in the pathology groups and available for clinicians and pharmacists as well as top management via intranet tools. 

## 3. Results

We present data from 2019. 

### 3.1. Cross Monitoring

The focus of this activity is to evaluate and monitor the trend of expenditure of production factors. This is the first step to a more in-depth analysis of the reasons for any budget overrun. 

The following tables (Table 1 and Table 2) report examples of cross-monitoring reports.

### 3.2. GDP Monitoring

Monthly, IRST analyzes expenditure trends for each GdP. Initial deviations from the budget are linked to variations in the number of patients or to different average costs per treatment (Table 3).

In case of variations in the average cost per patient, compliance with all the indicators of appropriateness and cost-effectiveness previously negotiated between the budget objectives for each GDP are re-evaluated.

#### 3.2.1. Off Label Treatment Monitoring

A monthly report is extracted regarding spending for Off Label use of drugs (Table 4). Research Institutes intrinsically consider therapeutic approaches using the concept of “precision medicine”, which in many cases is associated with an added patient value but not reimbursed by the NHS. Spending appropriateness is evaluated in these terms in off-labelling monitoring through a report that also underlines the part of expenditure covered by the AIFA 5% fund.

#### 3.2.2. Monitoring of Indicators of Appropriateness, Cost-Effectiveness and Operational Efficiency

The most sophisticated and in-depth level of monitoring is represented by the multidimensional set of indicators described previously, included in the budgets and objectives of the various operating units. The management systems and the data collected allow IRST to proceed to an assessment of prescription appropriateness, therapeutic adherence and/or ad hoc evaluations of off-label drug use, CNN (C-Non-Negotiated), etc., in every single case. The IRST assesses prescription appropriateness, therapeutic adherence and/or ad hoc evaluations of off-label drug use for each departmental unit according to the objectives defined in the negotiation phase. 

#### 3.2.3. Monitoring Appropriateness and Adherence to GReFO Recommendations

This continuous monitoring process is based on data available in electronically stored medical records.

Further monitoring, according to the recommendations issued by the regional group for each drug, is performed twice a year. ISRT calculates the expected consumption index for Forlì-Cesena according to the GReFO recommendations. Conformity assessments (compliant/non-compliant) are evaluated considering the GReFO recommendations (positive and/or negative, strong and/or weak), population indexes, specific cases treated in the IRST, registration times and drug availability (an example in available Appendix B) are also evaluated in the final report.

#### 3.2.4. MEAs Monitoring

**Figure 4:**
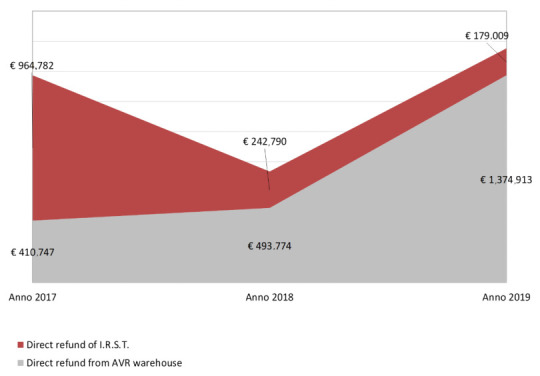
Trend of re-funds; Onco-AIFA years 2018–2019.

MEAs (Managed Entry Agreements [18]) are not considered during budgeting because their effectiveness is active only the following year. Yet, strict monitoring of this AIFA registry exists as it impacts the sustainability of the financial statement of the IRST. Figure 4 depicts MEA refunds from 2017 to 2019.

IRST can buy drugs both directly and through a centralized sub-regional warehouse of Area Vasta Romagna.

Once a year, a reimbursement procedure is requested only for drugs purchased from the centralized warehouse.

The costs avoided in 2019 (Table 5) were over 3 million euro for IRST and almost 2 million euro for the Romagna AUSL and for patients from outside the AVR (extra Area Vasta Romagna) of which IRST is directly responsible for budget management, while not benefiting directly in terms of resources. 

## 4. Discussion

In Europe, several countries have adopted a drug budgeting silo technique [19]. In France, Germany, Spain, the United Kingdom, and the Netherlands, even though national healthcare systems handle drug expenditure in different ways, at least once a clear case of “silo budgeting” has been applied and a national-level drug budget has been defined as a target. Only the United Kingdom, thereafter, abolished national drug-spending targets in favor of an integrated budget with local allocations for spending. Yet, in defining local budgets, each country adopts different systems. Spain and Italy mandated budgetary control to the regions maintaining national targets. In France [20], at the national level, budgets are set by the parliament based on GDP (Gross Domestic Product) growth, public sector deficits, and other macro criteria. The German government [21] established a national drug budget and allocated it among its 23 regions; each region has its own separate drug budget and a surplus in one region cannot be used to compensate for a deficit in another. A large variety of mechanisms were proposed for monitoring “over prescription” (i.e., regional drug budgets, drug budgets per physicians, copayment), but after initially positive results, the process was found to be ineffective.

The new funds for innovative oncological drugs in the Italian national and regional health system use innovation with reduced time, improve patient access and allow better planning capacity than methods based on historical expenditure; all positive and ameliorative aspects. This process allows homogeneity in the use of innovative drugs among different territories. 

On the other hand, drug expenditure for oncological drugs, based on the two available funds on a national and regional level, have the following limits:

- Establishment of expenditure caps without considering the number of patients and patient case-mix which encourages potential inappropriate behavior by providers who exceed maximum expenditure during the year;
- Management by non-communicating “silos” (fund for direct purchases, territorial pharmaceutical fund, funds for oncological and non-oncological innovation, etc.) that does not consider compensation between funds.

The innovative and positive aspects may prevail over the potential drawbacks if two conditions are met:

1. Programming is as well calibrated as possible (based on concurrent epidemiological data, needs and appropriateness);
2. A certain degree of flexibility is allowed during the year according to unforeseen events, such as different patient flows between the various providers, new clinical evidence, new therapies available.

If these conditions can be satisfied, the programming tools applied to the funds for innovative drugs should be extended to all high-cost oncological drug spending, which requires refined, evolved and complex governance tools. The generally accepted budget assessment based on historical spending should be replaced by a method that allows production factors analysis governed by consumption evaluations and expenditure weights per capita weighted expenditure, commonly used for territorial pharmaceutical spending.

## 5. Conclusions

In conclusion, the correct management of health expenses in the last years has become key in all European countries; defining value-based healthcare models to correctly allocate economic resources means taking care of patients more appropriately and with the latest available technology.

In the complex setting of oncological drug management, in which limited and insufficient public resources are available, the use of digitalized, multi-factorial analysis and decision-making alternatives may aid in sustainable management.

In order to do that, it is necessary to use sophisticated tools (computerized tools) that allow a detailed, periodic, and timely monitoring of a complex system of a defined set of measures and indicators of needs, appropriateness, and expenditure, in line with healthcare services demand. The IRST of Meldola, organized in a matrix based on pathology groups (GdPs), has developed over the years advanced tools for the analysis of needs, consumption, and expenditure of oncological drugs, specific for each disease, with a budgeting model focused on its determinants (appropriate and inappropriate) rather than on expenditure and measured with a set of indicators set based on the objectives of each UO (Operating Units). Thanks to this model, IRST has made the most out of the management innovations described above also in the dialogue with the regional authorities, promoting their implementation. A significant evolution in the governance of oncological pharmaceutical expenditure has great potential to succeed. 

These tools will ensure universal and timely access to therapeutic innovation in the field of oncology.

The model of IRST represents a way to allocate economic resources in taking care of patients, quality of care and support hospital structures in considering and including in decisions (economical and healthcare related) all information derived from institutional and clinical sources and represents an answer to the need of respect in national and regulatory directives in terms of health expenditure but also a way to address decisions in a value-based way through an accurate needs and scenario analysis. The proposed model attempts to provide a contribution for the governance of oncology drugs according to the described environment. 

## Figures and Tables

**Table 1:** Cross monitoring report of national and regional funds for innovative oncology drugs—Fund A.

Medicinal Products that Access the Innovation Fund (National)	Innovativeness Expiration Date (Date)	Effective Expenditure (€)	Reimbursed Expenditure (€)
Alectinib	31 January 2020	189,088	33,808
Daratumumab	31 December 2019	841,383	725,243
Nivolumab	24 March 2019	1,043,568	857,877
Atezolizumab	24 March 2019	134,980	128,702
Pembrolizumab after 1st line	10 May 2019	894,781	2,298,835
Pembrolizumab 1st line (75%)		1,701,010	
Durvalumab	6 September 2022	6536	11,033
Citarabina and danorubicina	18 June 2022	20,459	13,742
Lutathera from June to Dec	29 March 2022	587,301	587,301
Total use group A		5,419,105	4,656,541
Fund group A			5,079,087

**Table 2:** Cross monitoring report of national and regional funds for innovative oncology drugs—Fund B.

Medicinal Products that Access the Innovation Regional Fund Emilia Romagna	Innovativeness Expiration Date (Month)	Effective Expenditure (€)	Reimbursed Expenditure (€)
Pembrolizumab after 1st line		567,003	495,381
Nivolumab	from April 2019	3,092,797	2,789,277
Atezolizumab	from April 2019	332,742	307,630
Ibrutinib		907,200	847,553
Nabpaclitaxel		378,296	342,783
Crizotinib		236,147	240,999
Lenvatinib		38,429	38,076
Palbociclib		337,602	319,007
Ribociclib		51,214	35,508
Abemaciclib		50	0
Carfilzomib		307,314	278,328
Osimertinib		303,550	283,977
Idelalisib		94,757	88,977
Pomalidomide		30,361	30,361
Total consumption group B		6,677,463	6,097,855
Fund group B			5,621,176

**Table 3:** Pathology Group (GdP) Budget monitoring report for 2019.

**GdP**	**Budget 2019** **(€)**	**Expected** **Var. % vs. 2018** **(%)**	**Effective** **Expenses 2019** **(€)**	**Effective** **Var. % vs. 2018** **(%)**
Immunology	2,282,843	+7.3	2,685,101	+26.2
Hematology	7,819,552	+25.8	6,656,601	+7.1
Breast	4,964,118	−2.9	4,150,834	−18.8
Gastroenteric	2,845,447	+4.3	2,789,128	+2.2
CDO	1,599,889	+84.2	966,957	+11.3
Thoracic	5,382,282	+3.8	5,798,600	+11.8
Urogynecological	2,940,570	+9.5	3,029,271	+12.8
Total	27,834,702	+11.7	26,076,492	+4.6
**GdP**	**Mean Cost 2019** **(€)**	**Mean Cost 2018** **(€)**	**B. exp. Change** **(%)**	**Obs. Change** **(%)**
Immunology	21,481	22,400	−18.0	−4.1
Hematology	13,984	13,401	+11.0	+4.4
Breast	6122	8064	+6.0	−24.1
Gastroenteric	4506	4511	+11.0	−0.1
CDO	5171	4236	+26.0	+22.1
Thoracic	11,505	11,031	+26.0	+4.3
Urogynecological	5859	5359	+26.0	+9.3
Total	8396	9359	+8.0	−10.3
**GdP**	**Pts 2019** **(*n*)**	**Pts 2018** **(*n*)**	**Absolute Variation** **(%)**	
Immunology	125	95	+24%	
Hematology	476	464	+2.5%	
Breast	678	634	+6.5%	
Gastroenteric	619	605	+2.3%	
CDO	187	205	−9.6%	
Thoracic	504	470	+6.7%	
Urogynecological	517	501	+3.1%	
Total	3106	2974	+4.2%	

Note: The total number of patients may not correspond to the sum of all values since each patient may be affected by several pathologies.

**Table 4:** Prescriptive Exception report for GdP in 2019.

GdP	IRST 2019 (€)	5% AIFA Fund 2019 (€)	Total 2019 (€)	IRST 2018 (€)	5% AIFA Fund 2018 (€)	Total 2018 (€)
CDO-TR	66,186	76,257	142,443	44,507	9591	54,098
Gastroenteric	146,736	122,141	268,877	83,501	37,633	121,134
Breast	90,856	4480	95,336	130,089	13,440	143,529
Uroginecology	157,163	0	157,163	147,661	5603	153,264
Lung	121,462	0	121,462	290,745	0	290,745
Hematology	166,914	19,311	186,225	189,861	28,824	218,685
Melanoma	0	0	0	0	0	0
Total *	749,317	222,189	971,506	886,364	95,091	981,455

Note: * no radiopharmaceuticals& immunoglobulins included.

**Table 5:** Costs Avoided report year 2019.

GDP	Clinical Trial 2019 (€)	UT 2019 (€)	Total Costs Avoided (€)
Genitourinary	1,009,487.07	172,045.61	1,181,533
Breast	70,813.20	0.00	70,813
Melanoma	1,230,228.56	154,879.78	1,385,108
Lung	806,145.30	264,419.19	1,070,564
Gastrointestinal	256,841.96	1,167.33	258,009
Rare tumors	174,060.74	40,780.42	214,841
Hematology	806,022.39	16,583.07	822,605
Total	4,353,599.22	€649,875	€5,003,475

## Data Availability

The data presented in this study are available on request from the corresponding author.

## Abbreviations

The following abbreviations are used in this manuscript (the acronyms used to refer to the Italian version):

AFO	Hospital Pharmaceutical Assistance expenditure (effective expenditure)
AIFA	Italian Agency of Drugs
ASL-AUSL	Local Healthcare Agency
AVR	Area Vasta Romagna
CNN	C-Non Negotiated
CTR	Regional Therapeutic Commission
DBO	Data Base Oncology
DIT	Nursing and Technical Management
ERP	Enterprise Resource Planning
FDA	Food and Drug Administration
FED	Total Reimbursed Expenditure
GRADE	Grades of Recommendation, Assessment, Development, and Evaluation
IRST	Istituto Romagnolo per lo Studio dei Tumori “Dino Amadori”
IRCCS	Institutes of Hospitalization and Care with Scientific Character
RER	Emilia Romagna Region
GdP	Group of Pathology—Simple Departmental Structures
GReFO	Regional Group of Oncological Drugs
MEA	Managed Entry Agreements
NHF	National Healthcare Fund
NHS	National Health System
NSCLC	Non-Small-Cell Lung Cancer
OS	Overall Survival
PFS	Progression Free Survival
PTR/P	Regional/Provincial Therapeutic Handbook
UOBSC	Operative Unit of Biostatistics and Clinical Trials
UUOO	Operating Units
URTTF	Research and Innovation Department of Technology Transfer and Training
URP	Public Relations Office
YTD	Year to Date

## Appendix A

**Table A1:** Example of Budget report.

**Perspective of Patients and Stakeholders**				
**Objective A—Improve the Perceived Quality of Care and Prestige of the Institute**				
**1. Measure and Improve the Perceived Quality and Engagement of Patients**				
**Action**	**Index**	**Target**	**Weight**	**Inf. Spec**
To strive for high standards of perceived quality	% very satisfied (clear/disp)	≥70%	10%	
	% very satisfied with model taking charge	≥70%		20%
Developing humanization projects and Alliance with patients	number of patients to be sent to programs	≥40	10%	30%
	% new patients with first nursing visit	≥70%		50%
**2. Increase the Brand Value and the Ability to Attract Public and Private Funding**				
**Action**	**Index**	**Target**	**Weight**	**Inf. Spec**
Develop the ability to apply to national and international notice and improve the success rate	No. of projects supported by external funding	≥3		
	
	N.ro of project submitted to notices/tender	≥3	10%	
**Objective B—Progressively Implement the Comprehensive Cancer Care & Research Network**				
**1. Systematize and Manage Intercompany Integrated Clinical Pathways (ICP)**				
**Action**	**Index**	**Target**	**Weight**	**Inf. Spec**
Existence ICP to be formalized		within 30/06/20		
Formalization and monitoring of KPIs to improve ICP	Number of ICP monitors	≥1	10%	
**Internal Processes Perspective**				
**Objective C—Pursue Operational Excellence and Economic-Financial Sustainability**				
**1. Monitoring and Improvement of Outcomes and Value of Oncological Treatments**				
**Action**	**Index**	**Target**	**Weight**	**Inf. Spec**
Care timing: monitoring and improvement of waiting time and clinical path (Epic, Radiology, Radiotherapy, Hospitalizations)	% pts with start of chemotherapy <60 days after surgery	100%		
	% pts with RT start < than days from intervention	100%	10%	
Improvement of appropriateness drug use indexes (GREFO, Biosimilars) and increase of studies with free drug supply	Adhesion rate to GReFO	≥90%		
	% in III CR line of Trifluridine vs. Regorafenib	≥80%		
	% biosimilar use on Bevacizumab	TBD	20%	
**2. Scientific Relevant and Good Quality Production (Compliance with IRCCS AAA Requirements)**				
**Action**	**Index**	**Target**	**Weight**	**Inf. Spec**
Achieve 2019–2020 scientific production objectives	IRCCS points	≥100	20%	
**3. Enhancement of Clinical Trial Activities and Increase in Recruitment Rates**				
**Action**	**Index**	**Target**	**Weight**	**Inf. Spec**
Increase the enrollment rate	in-office therapies started/therapies started		10%	

## Appendix B

The report is an example of the evaluations of IRST activities versus planned following GReFO recommendations. GReFO group plan number of patients to be treated for 100,000 inhabitants. IRST rebate this number in line with population of its territory and monitor the number of patients treated as reported in the following example.

**Table A2:** Example of GReFO recommendations monitoring for GdP Toracic year 2019—Lung NSLC.

				Consumption Index		Number of Patients Treated in I.R.S.T.			
Molecule	Recommendation	Expected Cases Consumption Index Forecast for Emilia Romagna Territories	Note	per 100,000 Inhabitants Planned by GReFO	Planned for Territory of Forlì-Cesena	Forlì- Cesena	Romagna	Extra Romagna	Evaluation
Durvalumab	Strong positive	140	Consolidation after chemoradiotherapy in pts non in PD con PDL1 ≥ 1%	3.1	12	3	1	0	Compliant
Pembrolizumab	Strong positive	300	I line PD-L1 ≥ 50%—non sq/sq	6.7	27	19	10	6	Compliant
Pembrolizumab + Pemetrexed + CDDP	Strong positive	600	I line PD-L1 < 50% o non noto NON SQ	13.5	53	0	0	0	Compliant
Pembrolizumab	Weak positive	540	II line WT (indipendent from histology)	12.1	48	3	2	1	Compliant
Nivolumab	Weak positive					12	0	2	Compliant
Atezolizumab	Weak positive					20	3	11	Compliant
Afatinib/Erlotinib/Gefinitib	Weak negative	35	NSCLC I line EGFR +	0.8	3	6	1	0	Not Compliant
Osimertinib	Strong positive	140	NSCLC I line EGFR +	3.1	12	6	4	2	Compliant
Nintedanib + Docetaxel	Weak negative	180	NSCLC II line WT	4.0	16	0	0	0	Compliant
Pemetrexed	Weak positive		NSCLC II line WT			3	0	2	Compliant
Docetaxel	Weak/strong negative		NSCLC + SCLC II line WT			8	4	3	Compliant
Erlotinib	NA		NSCLC + SCLC II line WT			0	0	0	Compliant
Osimertinib	Strong positive	60	NSCLC II line EGFR +, T790M +	1.3	5	2	0	0	Compliant
Docetaxel/TKI		50	NSCLC II line EGFR +, T790M not changed	1.1	4	1	0	1	Compliant
Ceritinib	Weak negative	3	NSCLC I line ALK +	0.1	0	0	0	0	Compliant
Ceritinib	Weak positive	20	NSCLC II line ALK +	0.4	2	0	0	0	Compliant
Crizotinib	Weak negative	3	NSCLC I line ALK +	0.1	0	0	0	0	Compliant
Crizotinib	Strong/Weak positive	12	NSCLC I line ROS-1 +	0.3	1	1	0	0	Compliant
Alectinib	Strong positive	52	NSCLC I Line ALK +	1.2	5	0	1	0	Compliant
Alectinib	Weak positive	20	NSCLC II line ALK +	0.4	2	2	0	0	Compliant
Docetaxel/doppietta platino		0	NSCLC II line ALK +	0.0	0	0	0	0	Compliant
Dabrafenib/trametinib	NA	/	NSCLC Braf V600+						
